# Families First: the development of a new mentalization-based group intervention for first-time parents to promote child development and family health

**DOI:** 10.1017/S146342361500016X

**Published:** 2015-04-01

**Authors:** Mirjam Kalland, Åse Fagerlund, Malin von Koskull, Marjaterttu Pajulo

**Affiliations:** 1 Title of Docent, Folkhälsan Research Center and Swedish School of Social Science, University of Helsinki, Helsinki, Finland; 2 Folkhälsan Research Center, Helsinki, Finland; 3 Folkhälsans Förbund, Helsinki, Finland; 4 Title of Docent, Academy of Finland and Finn Brain, University of Turku, Turku, Finland

**Keywords:** child development, family health, health promotion, mentalization, parental reflective functioning

## Abstract

**Aim:**

The aim of the present study was to describe the development of Families First, a new mentalization-based group intervention model for supporting early parenthood. The general aim of the intervention was to support well-functioning models of parenting and prevent transmission of negative parenting models over generations, and thus promote child development and overall family health.

**Background:**

In the Finnish society, great concern has aroused during the last decade regarding the well-being and mental health of children and adolescents. Increased number of divorces, poverty, substance abuse, and mental health problems among parents enhance the risk for child neglect and abuse. New effective, preventive, and health-promoting intervention tools are greatly needed to support families with young children. At present, the Families First intervention is being implemented in primary social and healthcare units all over Finland.

**Methods and findings:**

This article will provide a theoretical understanding of the importance of parental mentalization for the development of the parent–child relationship and the development of the child as well as proposed mechanisms of actions in order to enhance mentalizing capacity. The cultural context will be described. The article will also provide a description of the scientific evaluation protocol of the intervention model. Finally, possible limitations and challenges of the intervention model are discussed.

The aim of this article was to describe the development of an intervention model for supporting early parenthood. Developing an intervention model that seeks to promote family and child health as well as to prevent the subsequent development of mental health problems is a complex task. The Medical Research Council (MRC) in the United Kingdom has developed a stepwise framework for designing and evaluating complex interventions (Medical Research Council, 2000). The first step is pre-clinical or theoretical, and answers the question *why* this intervention ought to work. The next step answers the question *how* the intervention will work (i.e., what are the mechanisms by which the intervention seeks to induce change). The next phase involves conducting a pilot trial, followed by a definitive randomized controlled trial. The last phase is implementation. The original MRC approach has proved influential and is widely cited (Campbell *et al.,* 2000), but the need for further development soon became evident. Accordingly, a revised version of the MRC framework was published in 2008 (Craig *et al.,* 2008), the result of a need for a revision based on several papers that identified limitations in the original framework (Hardeman *et al.,* 2005; Oakley *et al.,* 2006; Campbell *et al.,* 2007). These papers also recommended greater attention to the early phase of piloting and development work, a less linear model of the evaluation process, the recognition that complex interventions may work best when tailored to local contexts, and the consideration of alternatives to randomized trials. Despite the scientific evidence that supporting parenthood as early as possible positively affects the health and development of the child (Olds *et al.,* 2007a, Olds *et al.,* 2007b), the programs are often deemed too time consuming and difficult to implement or to enter widespread practice (Farely *et al.,* 2009). In this article, we provide a theoretical understanding of the importance of parental mentalization for the development of the parent–child relationship and the development of the child. We propose mechanisms of action for enhancing parental mentalizing capacity through group-based interventions. Further, this article describes the cultural context and a justification for why a new way of working with parents is currently necessary in Finland. The article also describes the scientific evaluation protocol of the intervention model and discusses possible limitations and challenges of the intervention model.

## Family and child health in Finland

During the past decade, Finnish society has seen growing concern about the well-being and mental health of children and adolescents. The number of children in need of child welfare services or child psychiatric services has risen continuously. At present, about 80 000 (1.4%) Finnish children are in need of child welfare services, and over 18 000 children are placed into substitute care outside the home (National Institute for Health and Welfare, Finland, 2013).

In Finland, pregnant mothers are provided cost-free services during pregnancy in maternity clinics, and 99.8% of them use these services on average 10 times during pregnancy, including 97.8% of high-risk mothers (Kalland *et al.,* 2006). In practice, all first-time parents – both mothers and fathers – are invited to attend cost-free antenatal classes (Hakulinen-Viitanen *et al.,* 2008). After the baby is born, the family receives services and health check-ups for the infant in the well-baby clinics until the child is seven years old and school begins. Services at the well-baby clinics are free of charge for families, and the drop-out rate is <1% (Ministry of Social Affairs and Health, 2004).

Finnish maternity and well-baby clinics have emphasized mainly physical health, check-ups at key developmental milestones, and vaccinations. More recently, however, psychological well-being and the promotion of mental health have become key national targets. The new Finnish legislation governing the development of work in maternity and well-baby clinics places more emphasis on supporting the health of the whole family (Decree 380/2009), and first-time parents are to receive peer-group support.

The recent economic recession in Finland has forced reductions in costs for healthcare and social services. At the same time, unemployment and poverty rates are rising, and families are moving to urban areas in search of jobs, thereby weakening ties with friends and relatives. Some follow-up studies of the impact of cuts in social and healthcare services on child health and development during the economic recession of the 1990s in Finland show that cuts in basic services had a detrimental impact on children and led to growing numbers of children in need of mental health services or out-of-home placement (Somersalo, 2002; Leinonen, 2004). Effective and inexpensive early intervention tools are needed to avoid similar consequences from the current economic recession. In addition, preventive and health-promoting interventions are needed to support families with young children in general.

## The importance of early intervention

A positive parent–child relationship is important, as it enhances the overall development of the child toward healthier cognitive, mental, and social functioning (Cicchetti and Toth, 2009). Because the perinatal and early childhood period is a time of great psychological change for parents and a time when parental preoccupation and motivation to invest in their child are especially strong, early-phase parental support plays a key role in promoting beneficial parent–child interaction (Raphael-Leff, 1991; Leckman and Mayes, 1998; Slade, 2002). Accordingly, early intervention has proved effective in preventing problems in parent–child interaction, especially if the intervention coincides with the arrival of the parents’ first child (Olds *et al.,* 2007b). Furthermore, intervening is most effective among first-time parents, as not only does the current child benefit from the improved parental skills, but so may any additional children in the family.

## Parental mentalization and attachment security

The importance of mental representations during the perinatal period and early childhood has become an area of growing interest. Mental representations are internalized memories of experiences of interaction, of parenting, and being a child. These representations resurge strongly during pregnancy and early parenthood in both mothers and fathers (Ammaniti et al., 1995; Stern, 1995), and begin to shape the quality of the parent–child relationship from pregnancy onward. Negative, fragile, or idealized representations of one’s own childhood and childhood parenting experiences are significant in that they so easily affect the parents’ representations of their child at present and lead to misinterpretations of their child’s behavior, negative interaction experiences, and vicious negative interaction circles, thereby increasing the risk for child neglect and abuse (Pajulo *et al.,* 2001; Suchman *et al.,* 2005b).

In the context of early parenting, mentalization refers to a parent’s capacity to think about and understand their child’s feelings and experiences. Mentalization includes the ability to see the child as an individual, separate from the mother/parent, from early on. Parents with an adequate mentalization capacity are curious about what goes on in their child’s mind when he/she acts in a certain way. Importantly, the parent also evaluates his/her own experiences and feelings, and is able to consider both how they may affect the child and how the parent interprets the child’s behavior (Slade, 2002; 2005). Research has shown that a higher parental mentalization capacity associates with more sensitive child–parent interactions, more secure child attachment, and healthier child development (Grienenberger *et al.,* 2005; Slade *et al.,* 2005a). Studies have also found higher maternal mentalization skills to correlate positively with a child’s state regulation capacity, social skills, and ability to play and symbolize (Fonagy *et al.,* 2002; Fonagy, 2008). Despite its roots in the parents’ early childhood experiences, parental mentalization can improve in response to interventions that directly target reflective capacities in high-risk parenting (Schechter *et al.,* 2002; 2005; 2006; Slade *et al.,* 2005b; Suchman *et al.,* 2008; 2012; Pajulo *et al.,* 2012).

Because parental capacity to mentalize is considered a pre-requisite for parental sensitivity in parent–child interaction, it is also considered to be one of the factors facilitating secure child attachment (Fonagy and Target, 1997; 2006; Fonagy *et al.*, 2012). Although Fonagy and colleagues have referred to sensitivity as a core contributor to attachment security, they have also pointed out that attachment security does not improve solely through behavioral aspects of sensitivity. Rather, they suggest that attachment security is affected by the extent to which a child is treated as a psychological agent with his/her own intentions. In essence, the ability to treat a child as a psychological agent with feelings, desires, and thoughts different from those of the parent, who is interested in understanding these mental states, encompasses the definition of good parental mentalizing (Sharp and Fonagy, 2008). Parental mentalizing may also serve as a mediator in the intergenerational transmission of attachment security and play a key role in breaking a chain of ‘inherited’ at-risk models of parenting (Slade *et al.,* 2005a; Suchman *et al.,* 2012). Mothers with considerable experience of (early) deprivation and trauma, but who have, nonetheless, acquired sufficiently high mentalization capacity through corrective relationships, are more likely to have securely attached children than are mothers with early trauma and low mentalizing capacity (Fonagy *et al.*, 1991).

In short, the early parental capacity to mentalize well-enough is of specific importance in both research and clinical practice for at least the following reasons: (1) many of the derailments in a parent–infant relationship are rooted in pregnancy and in early parent–infant interaction (Slade *et al.,* 2009); (2) the focus on parental mentalization support in early parenthood implies a specific venue for preventive interventions and treatment; and (3) an intervention targeting first-time parents could have an especially powerful influence on neurobiological changes related to parental mental preoccupation with their own baby and parenthood (Mayes *et al.*, 2005). Thus, positive changes in early parental mentalization will likely reduce misunderstandings and lapses in parent–child communication, increase positive interaction experiences, and healthier emotional bonds between family members.

## Significance of group-based interventions

In recent decades, interest in group-based support for parenting has grown. Parental support can either target certain risk groups or serve as a universal support to parents in a normative population with no identifiable risk. Evidence suggests that closed, structured, and theoretically based group interventions may prove beneficial and have positive effects (Kalavainen *et al.*, 2007; Thomas *et al.*, 2007), especially when they target high-risk parents or parents with children suffering from behavioral disorders (Kazdin, 2003; DeGarmo and Forgatch, 2005).

However, strong arguments also favor open-access groups, particularly for parents expecting their first child. One advantage of a universal approach is the possibility to reach as-yet unidentified at-risk parents and parents with a suboptimal parenting capacity who would not participate in targeted programs. Studies show that the perceived health improves of parents who receive group support after the birth of their first child (Hanna *et al.,* 2002; Cox and Docherty, 2008). Through these peer-support groups, parents develop larger social networks, gain self-confidence, and obtain access to relevant information on child health and parenting (Hanna *et al.,* 2002). Because most studies have focused on mothers and their infants, active investigation of the impact of support groups on fathers’ interaction with their infants is lacking (Guest and Keatinge, 2009). More information is needed on the long-term effects of parental groups, including the effects of groups on fathers.

## Background of the current intervention model

This intervention is based on a format called Parents First, which was originally developed at the Yale Child Study Center (Yale University, CT, USA) as a 12-session group intervention. Parents First was the first intervention program developed to use a structured design to enhance parental mentalization (M. Goyette-Ewing *et al.,* 2002, personal communication). The program originally targeted families with toddler-age children in daycare to support parenting among at-risk families in urban city areas. The aim of the current project was to develop and adapt the original Parents First format for general family health promotion to the Finnish healthcare system. The adjusted Finnish format of the intervention protocol was named Families First. The aim was to develop an intervention that

∙ enhances parental mentalization capacity and sensitivity in parent–child interaction (dyadic approach);
∙ targets the whole family and promotes communication between family members, thereby creating conditions for healthy, secure relationships within the family (triadic approach);
∙ targets first-time parents with small children at no identifiable risk in order to build up sufficient resilience to withstand future adversities (health promotion approach);
∙ provides parents an opportunity to establish a strong social support network that will persist after the intervention is over (peer-support approach);
∙ can be integrated into basic municipal services as a universal support for all first-time families and which does not require personnel with extensive specialized therapeutic training (public health approach) to reach a large proportion of the population, while at the same time reduces expenditures and strain on special healthcare.

## Development of the Families First intervention

Development of the intervention began at the Folkhälsan Foundation for Health Promotion and the Folkhälsan Research Center [both non-governmental organizations (NGOs)] in Helsinki in 2007, and the development was carried out in two stages: a pilot phase followed by an implementation phase. At the same time, we developed and piloted the first research protocol for evaluating the impact of the intervention on family and child health (see Table 1). Between 2007 and 2009, the Families First intervention was piloted at four sites in seven groups. The first group leader training took place in Helsinki in 2009 with 16 participants, and the first Families First group arranged under municipal management began in 2010. In 2010, another NGO – the Mannerheim League for Child Welfare (MLL) – received funding for further development and nationwide implementation of the Families First program in collaboration with the Folkhälsan Research Center, the National Institute for Health and Welfare, the Ministry of Social Affairs and Health, and the Federation of Mother and Child Homes and Shelters in Finland.

**Table 1:** Process description of the development of the Families First group intervention

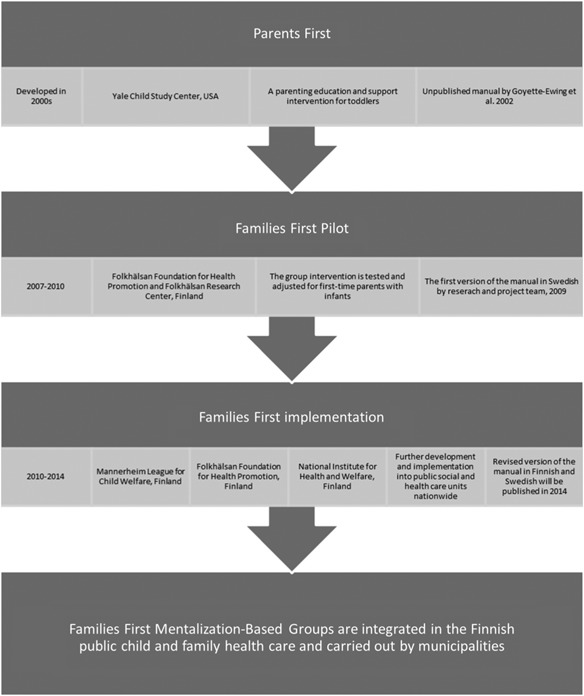

Two leaders (*n*=10) with group leader experience and a professional background in child health, social welfare, or education led all pilot groups, and the families were recruited from antenatal (childbirth) classes. Both parents participated with their children in the group meetings, which were held after office hours. The groups met 12 times, with the exception of one seven-session group, and no families quit any of the pilot groups. Four to six families participated in each group (total number of participants=37). Before the first pilot group, the leaders created an initial simple draft of a family group program. After every pilot group session, feedback was collected from participants through focus group interviews and/or questionnaires, and separately from group leaders. Based on the feedback from parents and group leaders, we carefully scrutinized the intervention to determine how well the various components supported parental mentalizing capacity and made adjustments accordingly.

## Changes in comparison to the original Parents First format

From the Parents First format, we incorporated into Families First the mentalization-based approach and the number of group meetings, as well as the themes and structure of most of the sessions; we also included ideas around inspiring questions, discussions, and homework, but only with adjusted age-appropriate content. In contrast to Parents First, which targeted and focused on working with parents/mothers while the children were invited to engage in other play activities, the focus of Families First became the whole family (mother, father, and child). To accommodate the Finnish public healthcare system, the leaders in Families First were employees of the municipality, and integrating the intervention was a part of their professional work. Therefore, a profession in mental health, considered important in Parents First, was not required. Further, the two group leaders held Families First sessions every other week, as opposed to one group leader holding weekly sessions in Parents First. In addition to the group sessions, the Parents First format included individual consultations as well as educational hand-outs on child development (M. Goyette-Ewing *et al.,* 2002, personal communication; Slade, 2002). In Finland, maternity and well-baby clinics already provide individual consultation and support to all families, and thus this element was omitted.

## Considerations and justifications for intervention content and structure

### Co-leading of groups

As a group leader, having a partner to reflect on group experiences proved to be useful in developing a reflective stance. Co-leading also provided a clearer view of the group processes and a means for modeling a mentalizing way of being together.

### Length of the Families First intervention

In accordance with the Parents First program and earlier studies related to the optimal length of structured group interventions that show a reduced effect if sessions exceed 16 (Bremberg, 2004), we determined that the Families First intervention would comprise 12 group sessions. The decision to proceed with a 12-session protocol was further supported by the experience of the one pilot group that met only seven times, as these parents had failed to adopt an independent mentalizing stance by the time the group session ended.

### Interval between gatherings

Weekly group sessions, as in Parents First, were considered too strenuous to implement within municipal basic services. Both parents and group leaders in the pilot groups were satisfied with group meetings every other week. The interval between meetings gave parents time to reflect on the themes raised during the previous meeting. The interval also meant that the child had time to develop and learn new skills, giving them new episodes to reflect on during the following meeting. When the Families First group sessions ended, the children were usually nine- or 10-months old, and several parents reported that the intervention had helped them notice and become more curious about their child’s physical, mental, and social developmental progress. In Finland, either parent is eligible for parental benefits until the child is almost one-year old; therefore, the period in which the family participates in a Families First group is also the time when at least one parent cares for the child at home. This provides excellent opportunities for the parent to observe the child and his/her own reactions as they interact.

### The importance of participation by the whole family

Right from the start, the importance of both parents’ participation became obvious. Just hearing their spouse talk about experiences in relation to their child deepened the parents’ understanding of each others’ perspectives and inner mental states. Their understanding of their child’s inner mental states also deepened when the parents reflected on their shared experiences, such as home activities. In some of the groups, the group leaders noted signs of parental competition for the child’s favor (triangulation). This changed as the group progressed, as the parents’ respect for their partner’s relationship with the child increased. The parents also began to talk about themselves more as a family than as two dyads (i.e., *me+the baby* or *you+the baby* became *we as a family*), while at the same time showed more appreciation for each family member’s uniqueness as a separate individual. This kind of triadic mentalization work may, in fact, prove more favorable for healthy, secure relationships within the family than supporting dyads within the family. It may even call into question whether reinforcing the dyadic relationship between only one parent and the child may lead to ignoring or excluding the other parent.

### From an educational approach to a ‘not knowing’ stance

At first, we decided to begin each session with a short introduction and handouts about an important issue related to child development, such as the child’s temperament or play, as we figured it would be easier for the parents to mentalize about their own child’s experience and feelings if they heard a theoretically oriented introduction as a starting point. However, in the first pilot group, parents either lost interest in what was happening within the group or reacted with open resistance to the more theoretical instructions; it seemed as though the level of abstraction alienated the parents from their own child and the lives they lived together. In the Families First intervention, the solution became to introduce each theme verbally at the beginning of each session, but to keep the main focus on a reflective dialog within the group.

### The group as a mentalizing setting

In response to experiences from the pilot group sessions, the arena for reflective work developed around concrete recent episodes from the parents’ everyday lives with their baby. In all pilot groups, some parents easily found specific moments about which to mentalize and subsequently inspired others through their reflections. A key insight from the pilot group sessions was to rely on the group’s ability to encourage mentalization.

### Facilitating parental mentalization through reflective questions

To enhance the mentalization process, we included reflective questions for each group session to serve as tools/guidelines for the group leaders and to inspire parents to take a closer, inquisitive look at their own child and at themselves as parents. The parents were supposed to find the questions fresh and challenging enough to encourage them to take a closer look and make an effort to look beyond the obvious. At the same time, questions had to be respectful and give parents the opportunity to control how much they wanted to share with the group. Based on the experience during the pilot phase, the structure and content of the Families First intervention was described in a manual (Folkhälsans Förbund, 2010; The Mannerheim League for Child Welfare, 2012). This manual presents mentalization theory and specific techniques to enhance parental mentalization capacity and reflective discussions within a group, as well as specific instructions regarding how to implement all 12 sessions. A final version of the manual will be published in 2014.

## Current structure and content of the Families First intervention

Families with first-born children can join a Families First group when their child is about three- to four-months old (tested time-span two to six months). The sessions are free of charge for the families. Both the mother and the father as well as the baby from each family are encouraged to participate, and single parents are also welcome to participate alone or with a support person. As noted above, two trained group leaders, forming a working couple, together lead each group. A maximum of four to six families can participate, making a total of 12 to 18 participants, plus the two group leaders.

The group is to meet in a calm and child-friendly environment, usually in the evening to enable parents to participate after work. Each gathering lasts ~2 h. During the first half-hour, families settle down to a light free meal and open discussion. The group then gathers more formally for the theme and discussion of the day. First, families are invited to talk about their homework activities followed by the actual theme of the day; finally, participants receive their new homework activity. The session ends with some informal time for feeding, changing nappies, and preparing to leave. Once the group has begun working, it is closed to new participants in order to promote trust between the participants and a willingness to share difficult or embarrassing experiences.

As the overarching goal of the intervention was to enhance parental mentalizing capacity, emphasis falls on reflections from the baby’s perspective, including the baby’s feelings, intentions, and needs. Using experiences from every-day situations such as nursing, bathing, or soothing a crying baby encourages parents to describe such moments in detail. With the concrete situation in mind, parents are then asked to stop and reflect on their own thoughts and feelings as well as what feelings or state of mind their baby might have experienced and expressed through his/her behavior. Parents are further encouraged to reflect on how their baby’s behavior influences them and how they might influence their baby in a mutual ongoing interaction. Parents may also be encouraged to reflect upon what they want for their own child at present or what their baby may be expecting of them. Group leaders facilitate and deepen their reflections with open questions such as ‘Tell us more,’ ‘How did you feel?,’ or ‘How do you think your baby felt at that moment?.’ By observing the coupling between their own mind and behavior, parents become more skillful at appreciating the mind behind their baby’s behavior and how they mutually influence each other. With time, group members increasingly pose reflective questions to each other, and thus help each other mentalize.

The sessions are designed in a format that progressively increases the demand on parental reflective capacity. In the first few sessions, parents are encouraged to observe their child closely, focusing on their child and his/her temperament, emphasizing every baby’s uniqueness, and how individuals and families may be uniquely different within the group. These initial discussions help to strengthen trust and the appreciation of differences between group members. Gradually, more challenging themes are introduced, requiring reflection on family members’ possibly different physical and emotional needs. Themes for discussions include the following: What do we wish for our children and how do we influence each other? How do we deal with strong, conflicting feelings in our children as well as in ourselves? How do we react to changes or help our children overcome adversities? How can we encourage our children toward increasing independence and resilience while acknowledging their need for dependence and safety?

Between group gatherings, parents are given home exercises that ask them to look at everyday episodes of interaction with fresh eyes and, in particular, to observe their baby’s reactions in relation to their own actions and mental states. These home activities can also serve an important function by encouraging families to do things together, all three of them, and to find pleasure in the little things in life as a family.

## Vignette: short episode from the 7^th^ group session

The previous time, the theme was ‘transition phases’, and all group discussions normally start with a look back at the previous session, but this time the group leader had no time to take the floor.

Mom: You ought to ask me about transitions today; ask me about what’s new for Sara

Group leader 1: Ok, let’s start today off by asking Sara’s family the first question. What’s happening in your family right now? Has anything happened since we saw you last time?

Mom: Sara learned to crawl on the day she turned six months old! (triumphant statement)

Group leader 1: Tell us more. What happened?

Mom: She just suddenly started crawling. She’d already been trying for a long time, but she’d never really got anywhere before.

Group leader 1: How did it feel for you guys?

Both parents in unison: Fantastic!

Dad: And I also felt really proud that our little girl could do that.

Mom: And she’s started to find it easier now, too. She likes being on the floor for longer now.

Group leader 2: What do you think she’s making of this new discovery? What do you think it means for her?

Mom: I think she feels life has become a lot more interesting and fun.

Group leader 2: What makes you think that?

Mom: I could see her eyes were shining, and she was looking around as if to say ‘What should I head for now and touch?’ And she made that funny sound, like she always does when she is excited.

Dad: She looked really happy and had a big, wide smile on her face. She’d probably been pretty frustrated before because she couldn’t get to where she wanted to go on her own. I probably didn’t really understand how frustrating it must have felt for her before. I just thought that she was an impatient child who did not want to be alone. But now I understand her better. She has seen that we move where we want and she also wants to do it. It is very understandable.

Group leader 1: Do you think she noticed that you thought this was a fantastic and proud moment.

Mom: Definitely! She looked into our eyes and I clapped my hands. And we both smiled. It was a wonderful moment. I wrote it down in the book I write things in. I’m happy that you (dad) were at home when it happened. I think Sara also was.

Group leader 1: Why is that?

Mom: Well, you know, because he pulled out the video camera, and you all know what a movie star Sara is – always enjoying her moment in the spotlight.

Everybody in the group laughs and someone else continues by telling about what has been going on in their family.

## Training and implementation

The group leaders are mostly staff already working in the municipal child healthcare or social work who take on leading Families First groups as part of their daily work, an arrangement that minimizes costs. In addition, the Families First groups readily become part of the municipal child and family healthcare. Contrary to merely dispensing advice or lecturing, group leaders take a facilitating stance that enables families to find their own solutions and answers through reflective questions and self-reflection together with the group. Group leaders, thus, act as facilitators who set the process of mentalization in motion. After each session, the group leaders meet to reflect on their own and the participants’ reactions. Group leaders in training also maintain a process diary in which they record their own thoughts and reflections about the group.

The Families First group leader training consists of theory and practical applications of parental mentalization and parental reflective functioning, including how to apply a mentalizing stance as group leaders. The training also includes general information about group processes and on how to direct groups, as well as how to structure sessions and practical arrangements on how to conduct a Families First group. The training lasts four days (2+1+1). In addition, while conducting their first Families First group, the group leaders must attend three compulsory days of supervision and recollection of key aspects of how to work with a mentalizing stance. Municipalities have received group leader training free-of-charge during project funding, but such trainings will be subject to charge from 2015 onward.

## Implementation to date

Implementation of the Families First intervention program is a joint collaboration between the MLL and the Folkhälsan Foundation for Health Promotion (Folkhälsan). Finland is a bilingual country, with both Finnish and Swedish as official languages. Co-operation between organizations functioning mainly in either language (MLL in Finnish and Folkhälsan in Swedish) enables the model to spread across the country to both language groups. At present (2014), about 80 municipalities all over Finland have signed a written contract with MLL to participate in the Families First intervention. Through the contract, the municipality agrees to have MLL or Folkhälsan train relevant local community healthcare professionals in the Families First method and subsequently carry out the Families First intervention for first-time parents in their municipality. In addition, municipalities also agree to participate in a research evaluation of the intervention. Eventually, we hope to offer cost-free Families First groups to all first-time Finnish families through the maternal and well-baby clinics.

Between 2010 and 2013, 222 groups were held for 967 families with a total of 2782 participants from different parts of the country. By spring 2013, about 300 group leaders had received FF training, and by the end of 2014 about 450 group leaders will receive training (Mannerheim League for Child Welfare, 2013). About half of the group leaders are healthcare nurses; the other professionals involved include social workers, pre-school teachers, nurses, and psychologists. One five-day training for the trainers has also been conducted (*n*=22).

## Evaluation of the Families First intervention

Because the Families First intervention targets parent–child relationships on a large scale in Finland and has the potential for important societal improvements on the well-being of children and their families, it is essential to objectively explore the effects and efficacy of the program. During the pilot phase of the intervention, a pilot study served to test the instruments. In addition, a research group at Folkhälsan Research Center performed the research evaluation in collaboration with the MLL and the National Institute for Health and Welfare (THL) in Finland.

The general aims of the Families First intervention are to reduce parental stress, to strengthen the parents’ social support network, and to reduce postnatal maternal depression. Related to the impact of improving mentalization skills are specific aims to support the early relationship between the baby and both the mother and the father, to improve the child’s social emotional development and health, and to enhance marital satisfaction. Accordingly, we hypothesize that parents who participate in the Families First intervention will experience less parental stress, less parental depressive symptoms, greater marital satisfaction, and a stronger sense of having an impact on one’s own life (sense of coherence) than control parents. We further hypothesize that children who participate in a Families First group will see improved somatic health and developmental progress than children in the control group. Both the *general* positive effects of the intervention groups as well as the *specific* effects mediated by enhanced parental mentalization capacity will result in positive outcomes. In other words, we propose that an improved parental ability to consciously reflect more on their own and on their child’s mental states and behavior will serve as a mediating factor promoting the child’s health and well-being.

The intervention will be evaluated through a matched control-group design that compares the outcome in a sample of 200 families participating in the Families First intervention with the outcome in 1000 control families receiving standard community care. Families will be recruited from municipalities that have agreed to offer Families First groups to families with first-born babies. Both the intervention and control groups will receive all the usual support and services from the maternity and well-baby clinics. Control participants will comprise families who did not participate in a Families First group, either because they were not offered the possibility or declined to participate in the intervention. A much larger number of control participants was enrolled in order to permit matching between the intervention and control groups. Comparative analyses between intervention and control groups at baseline will check for selection bias.

Participants are recruited from among public health nurses in all municipalities in Finland who have agreed to participate in the intervention and the study (*n*=about 80). The public health nurses inform potential participants about the study at one of their regular visits during the third trimester of pregnancy. The participants also receive written information about the study as well as informed consent forms and a prepaid envelope.

The same assessments will serve in both the intervention and control groups as well as for both mothers and fathers. Participants will complete the first assessment (baseline) during late pregnancy (gestational weeks 28–32) and then receive a short check-up after the birth of the baby (one month). The following assessments will take place at pre- and post-intervention time points (at three months and one year of age) with a follow-up at two years of age. The standardized assessments and research protocol to be used in the research part are presented in Table 2. The data collection will take place in the form of web-based questionnaires accessible through a personal code. Data collection, including follow-up, is currently under way and will continue until the end of 2016.

**Table 2:** Descriptions of measures and time points when used in the study

Measures	Authors	Description	Timepoints used
Parental Reflective Functioning Questionnaire	Luyten *et al*. (2009); Pajulo *et al*. (2010)	Measures parents’ capacity to mentalize about their unborn baby/baby/child and parenting	Baseline, pre-intervention, post-intervention, follow-up
Swedish Parenthood Stress Questionnaire	Östberg and Hagekull (2010)	Measures stress-related to parenthood	Pre-intervention, post-intervention, follow-up
Index of Marital Satisfaction	Hudson (1997)	Probes experienced satisfaction and support in the marital relationship	Baseline, pre-intervention, post-intervention, follow-up
Edinburgh Postnatal Depression Screen	Cox *et al.* (1987)	Covers symptoms of depression during pregnancy and postpartum	Baseline, pre-intervention, post-intervention
Center for Epdemiologic Studies Depression Scale	Radloff (1977)	Screening test for depression	Follow-up
Parental Bonding Index	Parker (1979)	Retrospectively measures adult recollections of parental behaviors and attitudes toward the individual in childhood. Considered a measure of the parent’s own childhood attachment	Baseline
Brief Infant Toddler Social Emotional Assessment	Briggs-Gowan *et al.* (2004)	Screens social–emotional development and competencies for children from 12 to 36 months	Post-intervention
Child Behavior Checklist	Achenbach and Rescorla (2000)	Measures problem behaviors in children	Follow-up
The Emotional Availability-Self Report	M. Biringen *et al.* (2002), personal communication	Assesses parental perceptions of emotional availability in the parent–child relationship	
Sense of Coherence Scale	Antonovsky (1979)	Measures sense of comprehensibility, manageability, and meaningfulness of one’s environment	Baseline, pre-intervention, post-intervention
Demographic questionnaire	Constructed for the study	Demographic assessment	Baseline
Pregnancy and delivery questionnaire	Constructed for the study	Pregnancy, delivery, and current health status of the baby	After delivery
Mentalizing in practice	Constructed for the study	Measures actual daily use of mentalizing	Follow-up

Baseline=pregnancy 28–32 weeks; after delivery=one month postpartum; pre-intervention=baby three months; post-intervention=baby one year old; follow-up=child two years old. At all time points, the family is asked about their family life, the child’s health, important life changes, and received support.

## Importance and societal implications of the Families First intervention

To summarize, the Families First intervention is based on a strong theoretical background supported by preliminary findings. Families First combines expertise and elements from several different disciplines (infant psychiatry, early education, psychology, social work, family therapy, public healthcare, and preventive work) and is important for several reasons. Families First is an innovative model of health-promoting work with first-time parents and their children, bringing new working tools to public healthcare. Thus, the Families First group intervention may improve the efficacy of preventive work in existing public services (e.g., maternity clinics, well-baby clinics). Second, the Families First intervention focuses on an early phase of parenting and aims to prevent the development of relationship disturbances with the current child and future children in the family. Third, the Families First intervention promotes health and well-being within and between all family members. The intervention may impact the way parents continue to share ideas and thoughts about their child as well as the way they value their own and other family members’ inner experiences and feelings. The intervention promises to diminish or prevent misunderstandings and relationship distortions between the parents themselves and between the parent and child.

Generally, training in mentalizing can also benefit healthcare workers in their daily work with families outside the Families First groups. Rather than giving answers or instructions, healthcare staff can use more reflective questions and contemplation. Together with the parents, healthcare workers try to understand the baby’s perspective and to reflect on the experiences behind the baby’s behavior and reactions.

Parental mentalizing is the art of reflecting on the mind behind a child’s behavior. If it is possible to influence the way parents listen to their children, observe and reflect on their reactions, and take their children’s needs into account, to what extent could it influence the well-being of the generation of children growing up? On a societal level, could it reduce costs related to stress, depression, child maltreatment, or divorce?

## Limitations and challenges

A general challenge related to evaluating an intervention is the stability and loyalty of the model; we cannot ensure that all the groups are conducted according to the training and the manual. However, fidelity in relation to complex interventions is seldom straightforward (Hawe *et al.*, 2004), as some interventions are designed to be adapted to local circumstances. In our model, sorting out the specific effects of mentalization-based groups on family health and well-being required a fixed curriculum. It is essential to begin with a clear idea of how much change or adaptation to the intervention protocol is permissible, and to record variations in implementation so as to assess fidelity in relation to the degree of standardization required by the study protocol.

Another challenge involves not only recruiting the families, but also ensuring their commitment to participate in the entire intervention and the evaluation research. Some municipalities easily found families willing to join the groups, but for others recruitment proved more challenging. Fortunately, however, once the group sessions began, few families quit. A general challenge related to dissemination: if the evidence supporting the intervention is sufficiently strong, how do we ensure that the intervention model remains alive and well and in active use, and who will be in charge of its upcoming nationwide implementation when the training of group leaders become subject to charge in 2015?

Some municipalities found 12 sessions to be too expensive, and therefore questioned the length of the intervention. Then again, are 12 sessions enough for an individual to implement new habits of reflection, understanding, and behavior? To what extent or for how long will the ordinary parent continue using a mentalizing stance in day-to-day interactions with the child? The important question of whether it is possible to make long-term significant changes in parental mentalization capacity with a relatively short intervention remains to be answered.
